# Validation of an infectious bronchitis virus GVIII-specific RT-PCR assay and first detection of IB80-like strains (lineage GVIII-2) in Italy

**DOI:** 10.3389/fvets.2024.1514760

**Published:** 2024-12-09

**Authors:** Matteo Legnardi, Francesca Poletto, Giovanni Franzo, Valeria Harper, Luca Bianco, Cristina Andolfatto, Angela Blanco, Mar Biarnés, Laura Ramon, Mattia Cecchinato, Claudia Maria Tucciarone

**Affiliations:** ^1^Department of Animal Medicine, Production and Health (MAPS), University of Padua, Padua, Italy; ^2^Private Practitioner, Imola, Italy; ^3^CESAC-Centre de Sanitat Avícola de Catalunya i Aragó, Reus, Spain; ^4^Department of Comparative Biomedicine and Food Science (BCA), University of Padua, Padua, Italy

**Keywords:** infectious bronchitis virus, IB80, Italy, molecular epidemiology, layer

## Abstract

Infectious bronchitis virus (IBV) is a pathogen causing respiratory, renal and reproductive clinical forms in chickens of all ages and productive categories. Its proneness to mutation and recombination gave rise to a plethora of variants differing in terms of pathogenicity, antigenicity, and distribution, with relevant implications for disease control, mainly pursued by routine vaccination, and diagnosis, requiring a steady update of molecular and serological methods. Among the most recent additions to the current phylogenetic classification, based on S1 gene sequencing, is the discovery of an eighth genotype (GVIII), further divided into lineages GVIII-1 and GVIII-2. GVIII-2, whose best-known representative is IB80, has been reported since 2015 in Europe, the Middle East and Asia. Most detections occurred in layers and breeders and were possibly associated to egg production drops. However, experimental reproduction of its pathogenicity could not be achieved. The significant genetic divergence of GVIII from other genogroups prevents its detection by many of the commonly applied biomolecular tests, hampering the understanding of its spread and impact. This study describes the validation of a GVIII-specific RT-PCR assay and its application to analyze samples collected from layer farms in Italy, where the presence of this genotype has never been investigated. The in-house assay proved highly reliable and allowed to establish the circulation of GVIII-2 in the country: between April and June 2024, 11 out of 84 flocks (13.1%) in 5 out of 24 farms (20.8%) tested positive. Phylogenetically, all Italian strains clustered together, whereas their identity with IB80 was 96.8–97.7%. Reproductive signs were reported in one farm and respiratory signs in another, whereas no clinical findings were recorded in the remaining positive cases. Although no definitive conclusions are possible on their prevalence and clinical relevance, the obtained results suggest that the presence of GVIII-2 strains in Italy is neither novel nor sporadic, highlighting the need to revise diagnostic approaches and shed light on the epidemiology of this novel lineage.

## 1 Introduction

Infectious bronchitis (IB) is a widespread, highly impactful disease affecting chickens of all age and productive types, which may manifest respiratory, renal and reproductive clinical forms with varying degrees of severity (1). It is caused by infectious bronchitis virus (IBV), a single stranded, positive-sense RNA virus belonging to the species *avian coronavirus*, genus *Gammacoronavirus*, family *Coronaviridae*. IBV is characterized by a high mutation rate, estimated at 10^−4^–10^−5^ substitutions/site/year (2), and is highly prone to recombination (3). These features are the basis of IBV remarkable heterogeneity, which is reflected by the substantial number of existing genetic types.

The current phylogenetic classification, proposed by Valastro et al. (4), is based on the gene coding for the Spike protein, and particularly on the amino-terminal portion encoding its S1 subunit. The focus on the S1 is motivated by its functional relevance, as it plays a pivotal role in determining antigenicity and viral tropism (5), and the significant genetic variability observed in the respective genomic region (6). Initially, six genotypes (GI-GVI) were defined, which could be further divided into 32 lineages (i.e. GI-1, GI-2, GII-1,…); subsequent additions were made, including the description of new lineages (GI-28, GI-29, GII-1) belonging to existing genotypes (7–9) and the establishment of a seventh (10, 11) and eight genotype (12).

Despite its recent description, GVIII has already garnered considerable scientific interest, due both to its divergent features and extent of circulation. The first strain belonging to this genotype was PA/1220/98 (13), identified in the US and circulating also in Canada, which was originally considered as a unique variant as it did not cluster into any identified lineage (4). The formal definition of GVIII was prompted by the description of additional strains by Domanska-Blicharz et al. (12) and Petzoldt et al. (14). These viruses, reported primarily in Europe, shared a higher genetic identity than with their American counterpart, leading Petzoldt et al. (14) to name the described strain “IB80”, because of its 80% similarity to PA/1220/98 within the S1 gene. Since 2015, IB80-like strains have been signaled in several countries, such as Poland, Belgium, France, Germany, the Netherlands, Lithuania, Belarus, Russia, Jordan, Saudi Arabia, Spain, Kazakhstan, Ukraine, and Philippines (14–17).

Due to the recency and overlap among these studies and to the relatively low number of available sequences, the division of GVIII into lineages is not well established yet. According to the current classification guidelines, a new lineage is defined when a minimum of three full S1 sequences obtained from at least two separate IB outbreaks form a monophyletic group and exhibit more than 13% and 14% uncorrected pairwise distances at nucleotide and amino acid level from other lineages (4). Whilst the distance between PA/1220/98 and IB80 is far above this indicative threshold, only a single PA/1220/98-like full S1 sequence, named IBV/Ck/USA/CA/21-1883, is currently available (18). Nonetheless, the existence of two separate lineages, with PA/1220/98 as GVIII-1 and IB80 as GVIII-2 prototype strains, does not appear in doubt from a practical perspective, and multiple authors already adopted this division (19, 20).

Both GVIII-1 and GVIII-2 strains have been detected primarily in long-lived birds, such as layers and breeders (12–14, 17), suggesting a preferential host tropism. Moreover, GVIII-2 has been associated to egg production drops rather than respiratory or renal forms (12, 14). So far, isolation attempts to substantiate these findings have proven difficult, but a strain known as D2860, closely related to IB80, has been recently characterized (21). D2860 was collected in the Netherlands in 2019 from six-months-old commercial white layers experiencing egg production losses. However, neither clinical signs nor lesions could be reproduced in experimental conditions in specific pathogen-free layers of different ages (21), questioning field observations. These aspects are not the only unresolved issues regarding IB80-like strains, whose high antigenic divergence, demonstrated by the low cross-relationship with other genotypes (21), also raises doubts about the protection granted by currently available vaccines.

Aside from sporadic molecular identifications, the circulation extent of GVIII strains is yet to be fully understood. From a diagnostic perspective, the low degree of genetic identity with other genotypes entails that GVIII is not amplified by most of the commonly implemented molecular tests targeting the S1. Dedicated assays would be therefore required, but their routine adoption is hampered by the added costs of implementation, limited number of available tests, and scarce awareness of these emerging strains. It is therefore of primary importance to establish reliable and cost-effective diagnostic methods that can be applied to conduct wider epidemiological surveys.

To this purpose, an in-house RT-PCR assay was designed and validated to specifically amplify GVIII strains. Thereafter, it was applied to samples collected from layer flocks in Italy, where the presence of this genotype has never been investigated, allowing the first identification of the circulation of IB80-like strains in the country.

## 2 Materials and methods

### 2.1 Development and validation of a GVIII-specific RT-PCR

#### 2.1.1 Positive controls

The S1 gene sequence of strain CK/DE/IB80/2016 (GenBank accession no. MT591566) was synthetized and cloned into a pUC57 plasmid by GenScript Biotech (Rijswijk, the Netherlands). After elution, the plasmid copy number/μl was calculated and serial 10-fold dilutions, ranging from 106 to 100 viral copies/μl, were subsequently prepared for assay optimization and analytical validation.

In addition, known GVIII-positive samples were sourced from the Center de Sanitat Avícola de Catalunya i Aragó (CESAC, Reus, Tarragona). These samples, consisting of eluates of either tracheal or cloacal swabs, were collected in 2021–22 from layer or breeder farms in Spain and already tested positive by RT-PCR employing the primers Palike483F and Palike913R designed by Domanska-Blicharz et al. (12).

#### 2.1.2 Primer design

Multiple candidate primer pairs were designed for the specific amplification of GVIII strains using the Geneious Prime software (Biomatters Ltd., Auckland, New Zealand). The database of target sequences included all S1 sequences of GVIII strains available in GenBank at the time of the study, whereas the off-target database contained representatives of all IBV lineages belonging to other genotypes.

#### 2.1.3 Extraction, RT-PCR, and results visualization

Nucleic acid extraction was carried out with the High Pure Viral Nucleic Acid Kit (Roche, Basel, Switzerland) following the manufacturer's instructions. All RT-PCRs were performed using the SuperScript™ III One-Step RT-PCR System with Platinum™ Taq DNA Polymerase kit (Thermo Fisher, Waltham, MA, USA) on an Applied Biosystems™ SimpliAmp™ Thermal Cycler (Applied Biosystems, Waltham, MA, USA). PCR products were loaded onto a 2% agarose gel added with SYBR Safe™ DNA Gel Stain (Thermo Fisher, Waltham, MA, USA) and ran for 20 min at 100 V, then results were visualized with a Gel-Doc XR System^®^ transilluminator (Bio-Rad, Hercules, CA, USA). Before and between experiments, samples were stored at −80°C.

#### 2.1.4 Development and optimization of the RT-PCR assay

A panel of serial plasmid dilutions was tested with all primer pairs, varying the temperature of the annealing phase (56°C, 58°C and 60°C) and the duration of the extension phase according to the estimated primer melting temperatures and amplicon lengths. The rest of the thermal protocol followed the kit producer's indications. Reagent volumes for each sample were as follows: 12.5 μl of 2X Reaction Mix; 1.5 μl of each 10 μM primer; 0.5 μl of SuperScript III RT/Platinum Taq Mix; 4 μl of biology-grade water; and 5 μl of target RNA. The primer pair and thermal protocol showing the highest sensitivity whilst minimizing non-specific amplification were selected for analytical validation.

#### 2.1.5 Analytical validation

The selected protocol was validated by evaluating its analytical sensitivity, specificity and repeatability. To this purpose, its limit of detection (LoD_50_) was determined by testing 5 times the serial 10-fold dilutions of the plasmid. Specificity was assessed by testing a panel of samples positive for IBV field or vaccine strains belonging to commonly encountered non-GVIII lineages, namely GI-I (Mass), GI-12 (D274), GI-13 (793B), GI-16 (Q1), GI-19 (QX), GI-23 (Variant 2), and GII-1 (D1466). Other common respiratory viruses, such as avian metapneumovirus (aMPV), Newcastle disease virus (NDV) and infectious laryngotracheitis virus (ILTV) were also tested. Repeatability and applicability in a real-life diagnostic scenario were determined considering a panel of 14 samples, which included 6 GVIII-positive Spanish samples as well as eight randomly selected diagnostic samples, consisting of respiratory or cloacal swab pools collected from layer or breeder farms in European countries. All 14 samples were first analyzed with the Kylt^®^ IBV-Variant IB80 commercial kit (AniCon Labor GmbH, Hoeltinghausen, Germany) on a MyGo Pro^®^ real-time PCR instrument (IT-IS International Ltd., Stokesley, United Kingdom), to confirm or establish the presence of IB80-like strains. The samples were then tested three times, with each RT-PCR being prepared on a different day by a different operator, and the Cohen's kappa coefficient (κ) was calculated.

### 2.2 Application of the validated assay for GVIII-monitoring

Following validation, the assay was implemented in the context of an epidemiological survey performed in the Laboratory of Microbiology and Infectious Diseases of the Department of Animal Medicine, Production and Health (MAPS) of the University of Padua (Legnaro, Italy). Besides searching for commonly encountered IBV genotypes using a generic RT-PCR based on primers XCE1+/XCE2– (22), samples coming from layer farms in Italy were analyzed with the GVIII-specific assay, which was carried out as previously established. Information regarding the farm location, age at sampling, and possible clinical signs were also retrieved.

Samples consisted of 10 tracheal or cloacal swabs, which were eluted into 1× PBS and processed as pools, each representing a separate flock. Nucleic acid extraction and molecular assays were performed as described above. Positive samples were Sanger sequenced using both primers at Macrogen Europe Milan Genome Center (Milan, Italy). Visual inspection and trimming of the obtained chromatograms were performed in 4Peaks (Nucleobytes B.V., Aalsmer, The Netherlands) and consensus sequences were generated in ChromasPro (Technelysium Pty Ltd., Helensvale, QLD, Australia). After aligning the detected strains with relevant reference sequences retrieved from GenBank, appropriate phylogenetic analyses were conducted using MEGA X (23).

## 3 Results

### 3.1 Development and validation of a GVIII-specific RT-PCR

#### 3.1.1 Development and optimization of the RT-PCR assay

Among the designed primer pairs, those with the best performance were IB80-672F (5'-CTAAGGGGGTTTTGGCTTGC-3') and IB80-1349R (5'-GTAACCACCGGATAAGGCCA-3'), which amplified a 676 nt-long portion of the S1 gene. The thermal protocol was set as follows:

- Retrotranscription: 50°C for 30 min;
- Pre-denaturation: 95°C for 2 min;
- 45 cycles of:
  – Denaturation: 95°C for 15 s;
  – Annealing: 56°C for 20 s;
  – Elongation: 68°C for 1 min;
- Final elongation: 68°C for 5 min.

#### 3.1.2 Analytical validation

In three out of five replicates, the lowest amplified dilution was 101 copies/μl, whereas the sample containing 100 copies/μl also tested positive in the two remaining occasions. The LoD_50_ was therefore equal to 101 copies/μl. Specificity assessment yielded perfect results, as the assay did not amplify any of the non-GVIII IBV strains nor the other respiratory viruses. Repeatability was also perfect (κ = 1), as the six Spanish GVIII-positive samples tested positive in each run, whereas the remaining eight samples were all negative. These results agreed with those of the IB80-specific commercial real-time RT-PCR kit, according to which the six Spanish samples were positive with Ct values ranging from 22.2 to 29.1, whereas the other diagnostic samples were negative.

### 3.2 Application of the validated assay for GVIII monitoring

**Figure 1:**
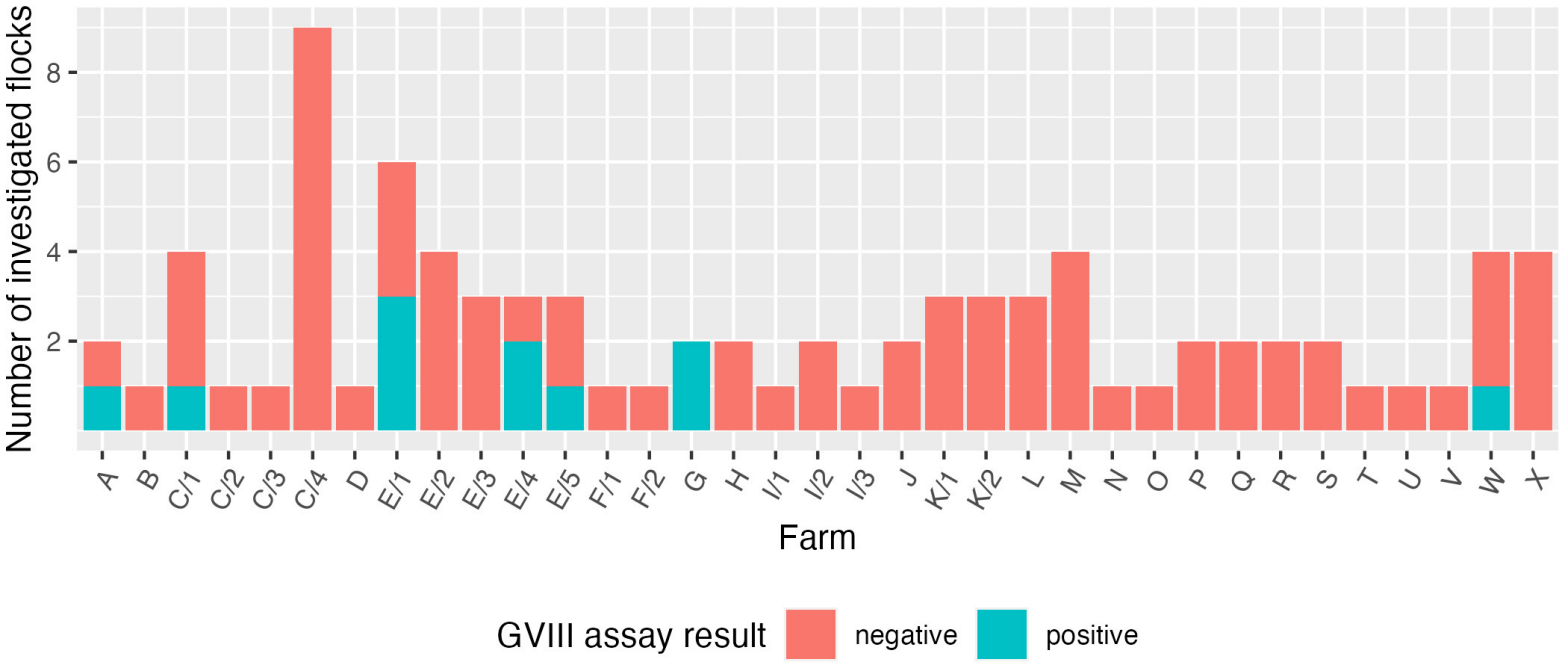
Results obtained using the GVIII-specific assay, divided by farm. Flocks from farms sampled multiple times were split accordingly.

From April to June 2024, samples were collected in 84 flocks belonging to 24 layer farms (some of which sampled multiple times) located in the Italian regions of Emilia-Romagna, Friuli-Venezia Giulia, Lazio, Lombardy, Piedmont, and Veneto. A total of 11 flocks (13.1% of the investigated ones) belonging to five farms (20.8%) tested positive to the GVIII-specific assay (Figure 1). The adoption of the generic RT-PCR also allowed the detection of other IBV strains: strains belonging to GI-13 (793B) were identified in 18 flocks (21.4%) from 12 farms (50%); 17 of them were identical to vaccine strain 4/91, whereas the remaining one was identical to vaccine strain 1/96. Moreover, strains belonging to GI-19 (QX) were detected in seven flocks (8.3%) from three farms (12.5%).

Among the GVIII-positive samples, J205 was one of the two specimens collected from farm B, where transient respiratory signs were reported. A 21-week-old asymptomatic group (sample J252) tested positive in multi-age farm C. Three flocks sampled at the same time point, and 11 sampled in three further occasions, all proved negative. Samples J333, J335 and J336 were collected from three flocks of multi-age farm E. The same farm was investigated four other times, with three samples collected at two separate time points testing positive (J985, J986, and K059). However, all flocks from farm E were asymptomatic. Samples J465 and J466 were taken from two flocks of farm G reportedly experiencing a drop in egg production as well as egg quality issues. Lastly, J863 was the only positive sample out of four collected from farm W, where no clinical signs were observed.

Five coinfections were identified between GVIII and other genotypes, two involving 4/91 strains and three with QX IBVs. More information about the GVIII-positive samples is provided in Table 1.

**Table 1:** Information about the samples which tested positive to the GVIII-specific RT-PCR.

**Sample**	**Farm**	**Sample type**	**Location (province)**	**Age (weeks)**	**Reported clinical signs**	**Generic RT-PCR result**	**GenBank accession no**.
J205	B	Cloacal	Mantova	37	Respiratory signs	Negative	PQ067232
J252	C	Tracheal	Verona	21	None	Negative	PQ067233
J333	E	Tracheal	Bologna	50	None	Negative	PQ067234
J335	E	Tracheal	Bologna	29	None	GI-13 (793B)	NA^*^
J336	E	Tracheal	Bologna	49	None	GI-19 (QX)	PQ067235
J985	E	Tracheal	Bologna	18	None	G1-19 (QX)	PQ067236
J986	E	Tracheal	Bologna	18	None	Negative	NA^*^
K059	E	Tracheal	Bologna	50	None	Negative	PQ067237
J465	G	Tracheal	Mantova	29	Drop in egg production, worse egg quality with pale eggshells	Negative	PQ067238
J466	G	Tracheal	Mantova	29	Drop in egg production, worse egg quality with pale eggshells	GI-13 (793B)	NA^*^
J863	W	Tracheal	Pordenone	30	None	GI-19 (QX)	PQ067239

^*^The sequence was not deposited since it was identical to others detected in the same farm.

**Figure 2:**
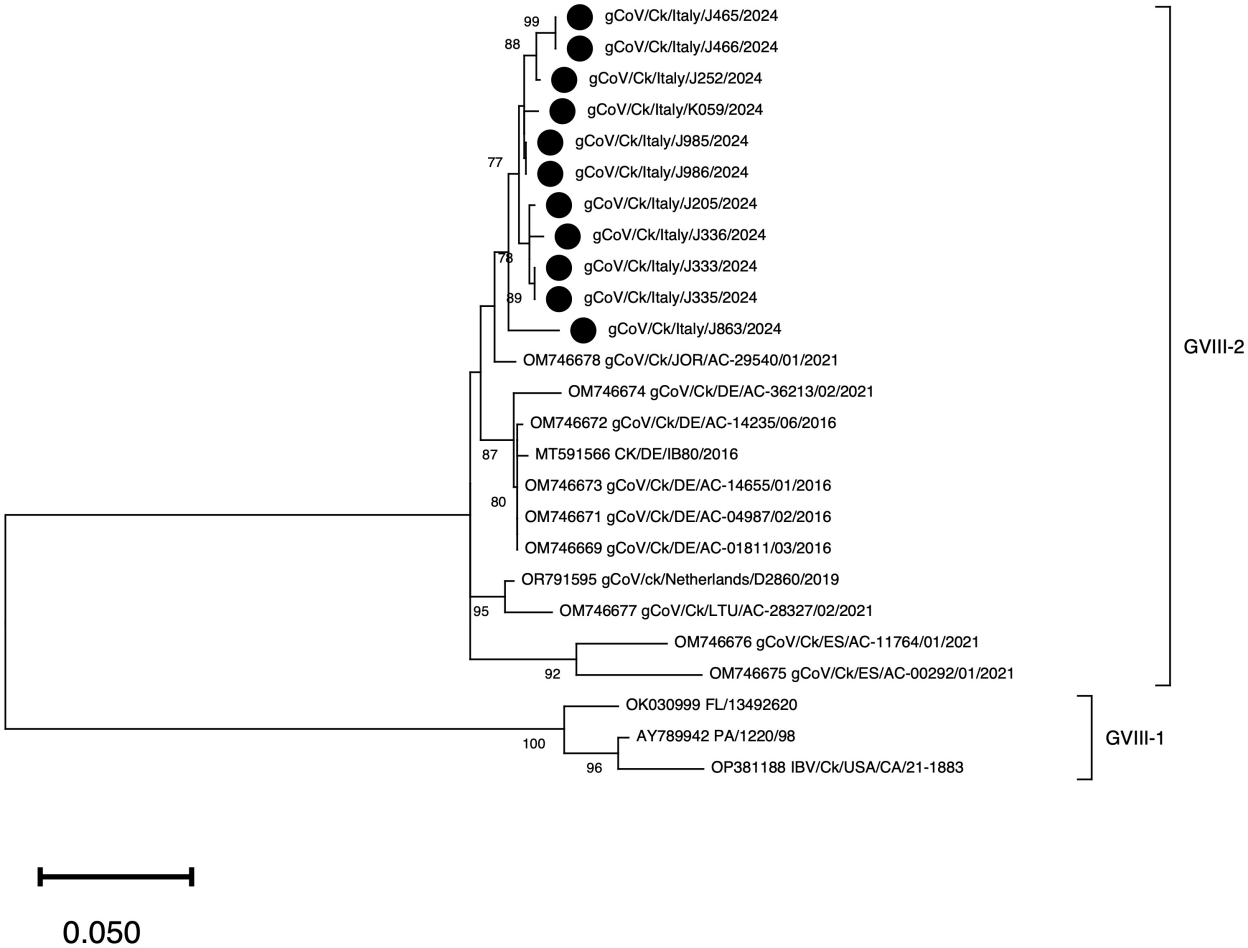
Phylogenetic tree of GVIII including the strains identified in the present study (marked with a black circle, •) and all available strains of sufficient length retrieved from GenBank. The tree was inferred with the Maximum Likelihood method (1,000 bootstraps) using the GTR+G substitution model (24), chosen according to the lowest Bayesian Information Criterion (BIC). Nodal support values are shown only when equal or higher than 70.

The 11 detected GVIII strains showed a reciprocal genetic identity ranging from 97.1% to 99.7% (excluding identical strains detected in the same farm), whereas the identity with prototype strain CK/DE/IB80/2016 was between 96.8% and 97.7%. A BLAST query yielded 20 sequences, of which only 14 covered the entire amplified S1 portion. The genetic identity with the detected strains was always above 81%, far superior to the 70% genotype threshold proposed by Valastro et al. (4). Phylogenetic analyses revealed that the Italian sequences formed a cluster within the GVIII-2 lineage, with the closest sequence being of Jordanian origin. Sequences from other European countries formed three robustly supported clades, one grouping German strains (including IB80), another made of a Dutch and a Lithuanian strain, and a third one comprising Spanish IBVs (Figure 2).

Among the characteristic nucleotide changes shared by all Italian strains, the T → C synonymous change at position 735 of the S gene was not found in any foreign GVIII-2 IBVs. At amino acid level, no novel changes were consistently observed in Italian sequences, although several non-synonymous mutations were unique to a subset of them (Supplementary Table S1). Most notably, compared to CK/DE/IB80/2016, all but one Italian GVIII-2 strains showed a D271G change; all Italian strains were different from the GVIII-2 prototype strain at position 388 (S388G/V); and more sporadic mutations such as L276V, T304I, N358D and H377Y were first noted in some of the sequenced strains.

## 4 Discussion

The presented results allowed to establish the presence of GVIII-2 strains in Italy for the first time. Albeit novel, this finding is not totally surprising, as Europe is the continent where the presence of this lineage has been best documented (14–17). Nonetheless, the low number of available sequences belonging to GVIII-2 (and even less to GVIII-1) is a clear indication that its actual extent of circulation is still unknown. This issue is more relevant for GVIII than other genotypes, as its high genetic divergence implies a poor diagnostic sensitivity if the implemented molecular diagnostic protocol does not account for it. Raising awareness toward its spread is thus crucial to prompt additional investigations and update the routine diagnostic tools, with cascading benefits for our understanding of GVIII-2 epidemiology.

Although the survey was based on convenience sampling, the multiple detections of IB80-like strains suggest a non-negligible presence in Italy. The positive farms were located in different provinces of three regions that include some of the most densely poultry populated areas of the country, supporting a wide, and possibly impactful, GVIII-2 circulation. To maximize the chances of detecting IB80-like strains, the present survey took into consideration only layer flocks, based on previous reports supporting the preferred host tropism of IB80 (14). This makes the GVIII-2 detection rate even more remarkable since the long productive cycle of layers lowers the odds of identifying IB infection if sample collection is not guided by clinical suspicion. On the other hand, the herein described results do not imply that GVIII-2 may not be circulating in the Italian broiler population as well, requiring dedicated investigations.

From a genetic perspective, all detected strains showed a high relative genetic identity and shared some characteristic nucleotide mutations, suggesting a single introduction event. Although not present in all Italian sequences, some unique amino acid changes were also noted in correspondence to the hypervariable region 3 (HVR3), a portion of the S1 that plays a critical role in antigenicity determination and receptor binding (25). Monitoring the local GVIII-2 evolution will be therefore important to ascertain whether these changes were sporadic or if they will become predominant in the local viral population, possibly leading to functional differences compared to other viruses belonging to the same lineage.

The evidence collected in this study on GVIII-2 clinical implications was not definitive: the infection proved unremarkable in most cases, whereas respiratory signs were reported in one farm and a negative impact on both egg quantity and quality was reported in two flocks from another one. However, it should be noted that drops in egg production, which seems to be the primary consequence of IB80-like infections (14) might be difficult to appreciate in multi-age layer farms (like several of those sampled), as eggs are collected together and issues limited to single flocks might be evened out.

Other IBVs, belonging to GI-13 (793B) or GI-19 (QX), were also detected, sometimes in combination with GVIII-2 strains. While the 793B sequences were identical to reference vaccine strains and were coherent with the vaccinations administered before the onset of production, QX strains were deemed of field origin. As a matter of fact, GI-19 has represented the main field genotype circulating in Italy for many years (26). Like IB80, QX viruses may have implications for egg production, particularly due to their association to the false layer syndrome (27). Nonetheless, this clinical form is caused by early infections, whereas the seven QX detections, including the three described coinfections with GVIII-2, all occurred during the laying period.

Aside from the results of the molecular survey, one of the main outcomes of this study was the validation of a molecular assay to enable the detection and characterization of IB80-like strains. The validated test demonstrated good performance in terms of analytical sensitivity, specificity, and reproducibility, and, according to the primer design, it would allow specific amplification not only of strains belonging to GVIII-2, but to the entire GVIII, finding potential use for broader investigations in diverse geographic contexts.

In conclusion, the present study lays the foundation for further research on GVIII spread and clinical impact, providing a reliable diagnostic option and insightful data on its circulation in Italy. Moreover, the reported findings help identifying knowledge gaps and limitations of current investigation strategies, stressing the need to define the actual moment of GVIII-2 introduction by analyzing retrospective samples and establish its prevalence in different productive categories and uninvestigated contexts through more structured epidemiological surveys.

## Data Availability

The datasets presented in this study can be found in online repositories. The names of the repository/repositories and accession number(s) can be found below: https://www.ncbi.nlm.nih.gov/genbank/, PQ067232 PQ067233 PQ067234 PQ067235 PQ067236 PQ067237 PQ067238 PQ067239.
