# Supplementary material for: Validation of an infectious bronchitis virus GVIII-specific RT-PCR assay and first detection of IB80-like strains (lineage GVIII-2) in Italy
Source: Front Vet Sci. 2024 Dec 9;11:1514760. doi: 10.3389/fvets.2024.1514760 (PMC11665066; doi:10.3389/fvets.2024.1514760)
Supplement: Supplementary file 1 [file Table_1.docx]

**Supplementary Table S1.** Comparison of amino acid sequences of available GVIII-2 strains within the S1 portion targeted by the validated assay (aa 225-445). Amino acid codes are reported when different from prototype strain CK/DE/IB80/2016. Italian strains are bolded.

|  | 237 | 238 | 244 | 245 | 253 | 256 | 257 | 262 | 271 | 273 | 276 | 277 | 279 | 281 | 301 | 304 | 310 | 323 | 327 | 331 | 341 | 357 | 358 | 361 | 363 | 364 | 371 | 373 | 377 | 378 | 380 | 384 | 385 | 387 | 388 | 390 | 394 | 410 | 411 | 416 | 420 | 428 | 438 |
| --- | --- | --- | --- | --- | --- | --- | --- | --- | --- | --- | --- | --- | --- | --- | --- | --- | --- | --- | --- | --- | --- | --- | --- | --- | --- | --- | --- | --- | --- | --- | --- | --- | --- | --- | --- | --- | --- | --- | --- | --- | --- | --- | --- |
| CK/DE/IB80/2016 | I | S | D | D | N | L | S | R | D | K | L | G | E | T | D | T | L | H | N | N | A | R | N | N | S | H | R | P | H | S | H | T | G | Y | S | S | I | Q | T | S | F | V | L |
| **gCoV/Ck/Italy/J205/2024** | . | . | . | **G** | . | . | . | . | **G** | **G** | . | . | . | . | . | . | . | . | . | . | . | . | . | . | **F** | . | . | . | . | **N** | . | . | . | . | **G** | . | **V** | . | . | . | . | . | . |
| **gCoV/Ck/Italy/J252/2024** | . | . | . | **G** | . | . | . | . | **G** | **T** | **V** | . | . | . | . | . | . | . | . | . | . | . | **D** | . | . | . | . | . | . | **N** | . | . | . | . | **G** | . | **V** | . | . | . | . | . | . |
| **gCoV/Ck/Italy/J333/2024** | . | . | . | **G** | . | . | . | . | **G** | **G** | . | . | . | . | . | . | . | . | . | . | . | . | . | . | . | . | . | . | **Y** | **N** | . | . | . | . | **G** | . | **V** | . | . | . | . | . | . |
| **gCoV/Ck/Italy/J335/2024** | . | . | . | **G** | . | . | . | . | **G** | **G** | . | . | . | . | . | . | . | . | . | . | . | . | . | . | . | . | . | . | **Y** | **N** | . | . | . | . | **G** | . | **V** | . | . | . | . | . | . |
| **gCoV/Ck/Italy/J336/2024** | . | . | . | **G** | . | . | . | . | **G** | **G** | . | . | . | . | **G** | . | . | . | **S** | . | . | . | . | **K** | . | . | . | . | . | **N** | . | . | . | . | **G** | . | **V** | . | . | . | . | . | . |
| **gCoV/Ck/Italy/J465/2024** | . | . | . | **G** | . | . | . | . | **G** | **R** | **V** | . | . | . | . | **I** | . | . | . | . | . | . | **D** | . | . | . | . | . | . | **N** | . | . | . | . | **G** | . | **V** | . | . | . | . | . | . |
| **gCoV/Ck/Italy/J466/2024** | . | . | . | **G** | . | . | . | . | **G** | **R** | **V** | . | . | . | . | **I** | . | . | . | . | . | . | **D** | . | . | . | . | . | . | **N** | . | . | . | . | **G** | . | **V** | . | . | . | . | . | . |
| **gCoV/Ck/Italy/J863/2024** | . | . | . | **S** | . | . | . | . | . | . | **S** | . | . | . | . | . | . | . | . | . | . | **K** | . | . | **L** | . | **K** | . | . | **T** | . | . | . | . | **V** | **K** | **V** | . | . | . | . | . | . |
| **gCoV/Ck/Italy/J985/2024** | . | . | . | **G** | . | . | . | . | **G** | . | **V** | . | . | . | . | . | . | . | . | . | . | . | . | . | . | . | . | . | . | **N** | . | . | . | . | **G** | . | **V** | . | . | . | . | . | . |
| **gCoV/Ck/Italy/J986/2024** | . | . | . | **G** | . | . | . | . | **G** | . | **V** | . | . | . | . | . | . | . | . | . | . | . | . | . | . | . | . | . | . | **N** | . | . | . | . | **G** | . | **V** | . | . | . | . | . | . |
| **gCoV/Ck/Italy/K059/2024** | . | . | . | **G** | . | . | . | . | **G** | . | **V** | . | . | . | . | . | . | . | . | . | . | . | . | **K** | . | . | . | **S** | . | **N** | . | . | . | **H** | **G** | . | **V** | . | . | . | . | . | . |
| gCoV/Ck/JOR/AC-29540/01/2021 | . | . | . | G | . | . | . | . | . | . | . | . | . | . | . | . | . | . | . | . | . | . | . | . | . | . | . | . | . | N | R | I | . | . | . | . | V | . | . | N | . | . | . |
| gCoV/Ck/DE/AC-14655/01/2016 | . | . | . | G | . | . | . | . | . | . | . | . | . | . | . | . | . | . | . | . | . | . | . | . | . | . | . | . | . | . | . | . | . | . | . | . | . | . | . | . | . | . | . |
| gCoV/Ck/DE/AC-04987/02/2016 | . | . | . | G | . | . | . | . | . | . | . | . | . | . | . | . | . | . | . | . | . | . | . | . | . | . | . | . | . | . | . | . | . | . | . | . | . | . | . | . | . | . | . |
| gCoV/Ck/DE/AC-01811/03/2016 | . | . | . | G | . | . | . | . | . | . | . | . | . | . | . | . | . | . | . | . | . | . | . | . | . | . | . | . | . | . | . | . | . | . | . | . | . | . | . | . | . | . | . |
| gCoV/ck/Netherlands/D2860/2019 | . | . | . | G | . | . | . | . | A | E | S | . | . | . | . | . | F | . | . | . | . | . | . | . | Y | . | K | . | . | T | R | . | . | . | . | . | V | . | . | . | . | . | . |
| gCoV/Ck/DE/AC-14235/06/2016 | . | . | . | G | . | . | . | . | . | . | . | . | . | . | . | . | P | . | . | . | . | . | . | . | . | . | . | . | . | . | . | . | . | . | . | . | . | . | . | . | . | . | . |
| gCoV/Ck/DE/AC-36213/02/2021 | . | . | E | S | . | . | . | H | G | . | . | . | . | . | . | . | . | . | . | . | . | . | . | K | . | . | . | Q | . | . | . | . | . | . | . | . | . | . | . | . | . | . | S |
| gCoV/Ck/LTU/AC-28327/02/2021 | . | . | . | G | . | . | . | . | A | E | S | E | K | . | . | . | . | . | . | . | S | . | . | . | G | . | . | . | . | T | R | . | . | . | . | . | V | . | I | . | . | . | . |
| gCoV/Ck/ES/AC-11764/01/2021 | V | A | . | N | . | . | N | . | N | . | S | Q | . | E | . | . | . | . | . | K | . | . | . | R | E | . | . | Q | R | . | R | Q | S | . | . | . | . | . | . | . | . | A | V |
| gCoV/Ck/ES/AC-00292/01/2021 | . | A | . | N | K | I | E | . | A | E | . | Q | . | K | G | . | . | P | D | . | . | . | . | Q | . | S | . | Q | . | . | R | Q | S | . | . | . | . | L | . | . | L | . | . |
